# SARS-CoV-2 Variants and Clinical Outcomes: A Systematic Review

**DOI:** 10.3390/life12020170

**Published:** 2022-01-25

**Authors:** Indira R. Mendiola-Pastrana, Eduardo López-Ortiz, José G. Río de la Loza-Zamora, James González, Anel Gómez-García, Geovani López-Ortiz

**Affiliations:** 1 Subdivisión de Medicina Familiar, Facultad de Medicina, Universidad Nacional Autónoma de México, Ciudad de Mexico 04510, Mexico; indira.mendiola@imss.gob.mx (I.R.M.-P.); eduardolptz@gmail.com (E.L.-O.); joseguillermoriodelaloza@hotmail.com (J.G.R.d.l.L.-Z.); 2 Departamento de Biología Celular, Facultad de Ciencias, Universidad Nacional Autónoma de México, Ciudad de Mexico 04510, Mexico; james@ciencias.unam.mx; 3 Centro de Investigación Biomédica de Michoacán, Instituto Mexicano del Seguro Social, Morelia 58351, Mexico; anel.gomez.garcia@gmail.com

**Keywords:** SARS-CoV-2 variants, mutations, clinical outcomes, outcome assessment, health care, severity, clinical presentations

## Abstract

Background: From the start of the COVID-19 pandemic, new SARS-CoV-2 variants have emerged that potentially affect transmissibility, severity, and immune evasion in infected individuals. In the present systematic review, the impact of different SARS-CoV-2 variants on clinical outcomes is analyzed. Methods: A systematic review was performed according to the Preferred Reporting Items for Systematic Reviews and Meta-Analyses (PRISMA) 2020. Two databases (PubMed and ScienceDirect) were searched for original articles published from 1 January 2020 to 23 November 2021. The articles that met the selection criteria were appraised according to the Newcastle–Ottawa Quality Assessment Scale. Results: Thirty-three articles were included, involving a total of 253,209 patients and 188,944 partial or complete SARS-CoV-2 sequences. The most reported SARS-CoV-2 variants showed changes in the spike protein, N protein, RdRp and NSP3. In 28 scenarios, SARS-CoV-2 variants were found to be associated with a mild to severe or even fatal clinical outcome, 15 articles reported such association to be statistically significant. Adjustments in eight of them were made for age, sex and other covariates. Conclusions: SARS-CoV-2 variants can potentially have an impact on clinical outcomes; future studies focused on this topic should consider several covariates that influence the clinical course of the disease.

## 1. Introduction

Variability in organisms leads to important changes which will have an effect on the course of their evolution [1,2]. In viruses, changes can determine their pathogenicity and virulence [3,4]; even single base changes can markedly influence their spread and confer selective advantages [5].

Since the beginning of the COVID-19 pandemic, it has been reported that SARS-CoV-2 has presented multiple changes in its genetic sequence that can potentially increase its infectivity, pathogenicity and antigenic capacity. This could affect the individual’s immune response and increase the severity of the clinical outcomes in each of the outbreaks [6,7]. One of the first variants to be recognized was D614G in the spike protein [6,8], and as genome sequencing subsequently progressed in different countries, it was reported that different mutations influence the adaptation of the virus to environmental and population contexts, in addition to conferring various phenotypes of clinical interest [9,10].

The clinical course caused by SARS-CoV-2 is associated with country-specific epidemiological and health contexts, age, pre-existing diseases, comorbidities, and host allelic variations [11,12]. However, meta-analyses and observational studies have shown that the so-called Variants of Concern increase the risk of disease severity and death, compared to other non-VOC variants, including the original Wuhan or “wild-type” variant [13,14]. This opens multiple questions about the interrelationship of the factors that condition the body’s responses to SARS-CoV-2 infection and emphasizes the need to study those variables that could impact the outcome of the infection; one question of importance is the interrelationship between variants of the virus and their clinical outcomes, an aspect that, due to the social, biological and methodological heterogeneity of the available evidence, has thus far not been explored in depth [13,14,15], hence the relevance of developing this systematic review.

## 2. Materials and Methods

The PRISMA 2020 (Preferred Reporting Items for Systematic Reviews and Meta-Analyses) guideline was used [16]. Due to the nature of this review focusing on the association between exposure and clinical outcomes, the PEO (Patient, Exposure of interest and Outcome) approach was used [17], which has been useful in other systematic reviews [18]. The question to be answered: what is the impact of SARS-CoV-2 variants on clinical outcomes in infected persons? A literature search was carried out of articles published from 1 January 2020 to 23 November 2021 in the PubMed and ScienceDirect databases. Advanced algorithm searches were performed using keywords with the use of Boolean operators. Originally, different search algorithms were considered which included the words: SARS-CoV-2 variants, SARS-CoV-2 mutations, Outcome Assessment, Health Care, Clinical Outcome, Clinical Output, Health Outcome, clinical profile, among others, but due to the limited number of articles available with different algorithms (*n* = 17 in some cases), the search was circumscribed as follows: ((SARS-CoV-2 Variants) OR (SARS-CoV-2 Mutations)) AND (Clinical Outcomes) for PubMed and “SARS-COV-2 Variants” OR “SARS-COV-2 Mutations” AND “Clinical Outcomes” for ScienceDirect.

A literature review was performed, and the available evidence on the topic of interest was condensed selected, compiled, screened, and analyzed independently by two authors (G.L-O. and E.L-O.). Articles focused on SARS-CoV-2 variants and mutations related to clinical outcomes were included. Review articles, essays, opinions, editorials, and case reports were excluded. Studies were analyzed according to changes in SARS-CoV-2 sequences and their clinical outcomes.

In a first approach, G.L-O. and E.L-O., independently analyzed titles and abstracts of 263 articles. After searching and filtering the information, 108 duplicated records were removed, later 107 were excluded after reviewing title and abstract due the articles were not related to the research question. Forty-eight articles were selected for complete reading to determine their relevance for the present review; 15 articles were subsequently removed, 12 because the SARS-CoV-2 variants were not related to clinical outcomes and 3 because the allelic variants were in humans. The controversies were resolved by discussions in which a third author participated (I.R.M-P.); 33 articles met the inclusion criteria.

The Newcastle–Ottawa Quality Assessment Scale checklists adapted for cross-sectional and cohort studies were used to assess the quality of the studies of the selected articles [19,20]. This systematic review was registered in OSF (10.17605/OSF.IO/3PM6Q).

**Figure 1:**
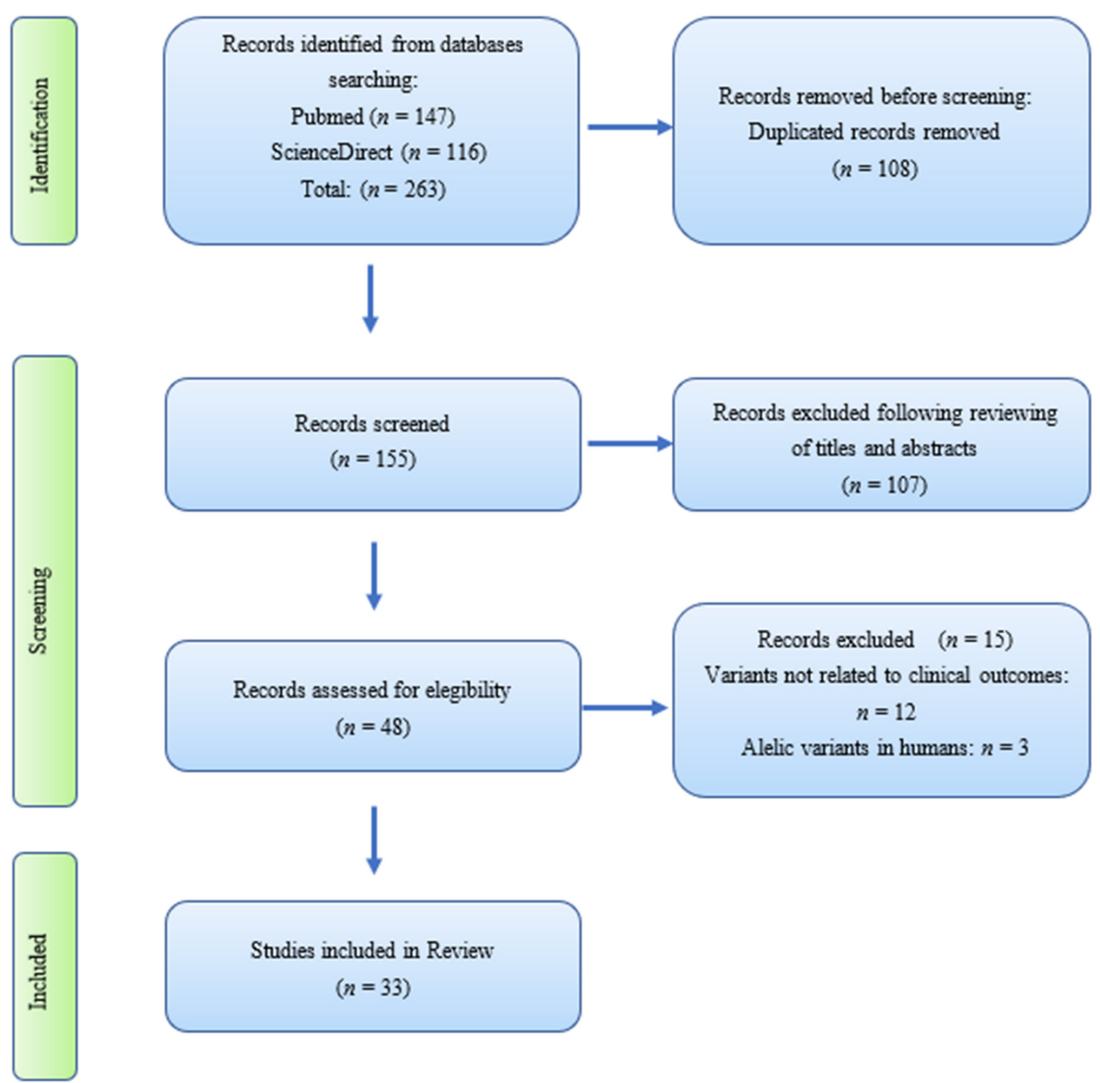
PRISMA flow diagram for search strategy.

Figure 1 presents the procedure used to select relevant articles according to the PRISMA 2020 guideline.

## 3. Results

There were 263 potentially relevant records identified in PubMed and ScienceDirect, 33 met the inclusion criteria. Data from 253,209 patients and 188,944 partial or complete SARS-CoV-2 sequences were analyzed in the referred studies. SARS-CoV-2 variants in the studies were grouped into clades, lineages, and others that were not referred within one of these categories, but by changes at the gene or protein level.

### 3.1. SARS-CoV-2 Variants

Once the articles were selected, the following step was to investigate SARS-CoV-2 mutations related to changes in the phenotype compared to the original virus. These mutations covered different levels of structural organization [21,22], but those that have been studied more are the ones that have an impact at the sequence level on the different viral proteins [6,23,24].

One of the first variants reported in the COVID-19 pandemic was D614G in the spike protein, which is associated with an increase in viral load, immune escape, possible drug resistance and increased pathogenicity. This amino acid substitution has been maintained in the different current variants. [21,23,24,25,26].

It has been pointed out that the region coding for the receptor binding domain (RBD) of the spike protein is prone to accumulate changes in SARS-CoV-2; 13 articles analyzed reported substitutions along this region, among them: N501Y, E484K, N439K, S477N, S399P, and K417V. It has been proposed that changes in this region could alter binding affinity of SARS-CoV-2 for ACE2 [11,13,15,27,28,29,30,31,32,33,34,35,36].

**Figure 2:**
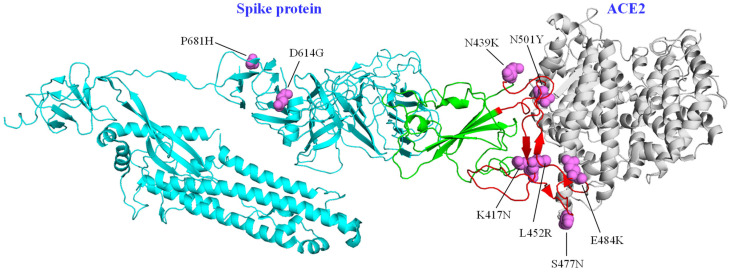
Main changes in spike protein reported in articles analyzed. ● Protomer of the spike protein; ● RBD; ● RBM; ● amino acid substitutions, ● ACE2 protein, (PDB structure [37,38], PyMOL v.4.6).

Another reported variant in the spike protein was P681H, which is located near the furin cleavage site and is associated with increased transmissibility and infectivity of SARS-CoV-2 [30,33]. The main Variants of Concern present changes in sequences associated to the spike protein, in the RBD and RBM (receptor binding motif) and the furin cleavage site. Some of the most relevant changes in the spike protein are illustrated in Figure 2.

The changes in SARS-CoV-2 are distributed in various sites in its sequence-like spike protein, N protein, RNA-dependent RNA polymerase (RdRp), NSP3, NSP4 and other open reading frames (ORFs) (Table 1).

### 3.2. SARS-CoV-2 Variants and Clinical Outcomes

SARS-CoV-2 variants can be classified by the statistical distribution related to phylogenetic groups. According to the Global Initiative on Sharing all Influenza Data (GISAID), there are eight clades related to specific genetic markers. Conversely, lineages have been classified according to genetic and epidemiological factors related to outbreaks in different geographic regions [46]. Although there are recommendations about the classification of variants, a significant number of the analyzed studies did not follow them. When analyzing the articles included in this systematic review, there was heterogeneity in classifying and defining variants, nine articles focused on clades, ten on lineages, and eleven on other variants, that were not referred to within these two categories, but by changes at the gene or protein level, two studies focused on clades and lineages, and one on clades, lineages and variants (Table 2).

When analyzing SARS-CoV-2 variants with their outcomes, ten articles reported that there was no relationship [23,25,26,31,34,36,40,42,43,47]; in six, there were possible associations between variants and clinical outcomes (from moderate to severe) [6,15,21,33,41,44], while in nine scenarios, mild clinical outcomes were reported [24,36,39,45,46,48], in fourteen severe outcomes [11,24,27,28,29,35,36,39,46,48,49,50,51], and in five fatal outcomes [13,22,24,30,32] (different outcomes were reported in more than one study). A statistically significant association was reported (from mild to severe/fatal) between variants and clinical outcomes in fifteen articles [11,13,22,24,27,28,30,32,35,36,46,48,49,50,51]. In eight of them, adjustments for age, sex, or other covariates were made [11,13,32,36,46,48,49,50] (Table 2).

Prior to the reporting of Variants of Interest (VOI) and Variants of Concern (VOC), changes in the SARS-CoV-2 sequence that could have an impact on clinical outcomes had been determined [6]. The D614G variant in the spike protein was initially considered to be related to a higher rate of hospitalizations and moderate to severe clinical outcomes [6,23]; however, analyses in different cohorts showed no relationship with disease severity; this change increases the adaptability of the virus in human populations, without necessarily causing more severe disease [25,43]. The same scenario was visualized for the N439K variant in the spike protein, which was also not found to have a direct effect on clinical outcomes, compared to the original virus. However, it was reported that this substitution had emerged in different clades independently and that it increased affinity for ACE2 and resistance against various neutralizing monoclonal antibodies [31].

A study determined that polygenic mutations in SARS-CoV-2 had different outcomes. For mild disease, the following amino acid changes were detected: L84S, G196V in ORF8 and ORF3a, respectively, as well as L37F substitutions in NSP6, F308Y in NSP4 and S197L in the N protein. When analyzing sequences of hospitalized patients, 15 changes distributed in seven genes were found: three in the spike protein, two in RdRp, two in ORF3a, five in N protein, one in ORF6 and two in NSP3; while in fatal outcomes, L71F changes were found in NSP7 and S253P in ORF3a [24].

In a study where associations between different mutations and clinical outcomes were analyzed, Zekri et al. [44] found in a sample of 50 patients that the V6 deletion in the spike protein was associated with an increased risk and duration of fever and nasal congestion, while the L3606-Nsp6 deletion was associated with an increased presence of cough and conjunctival congestion.

When variants with changes in P504L, as well as Y541C in NSP13 were analyzed, an association was found between these with infection and mortality rates, without correlation with other studies [41]. Likewise, the N501Y variant in the spike protein was found to have an increase, without statistical significance, of 18% in terms of risk of fatal outcome [34].

In silico studies have allowed for a proposal that there are mutation signatures responsible for promoting mild and severe outcomes, in which 20 mutations could be used to separate both groups. These are distributed in the gene encoding the spike protein, as well as in other viral proteins and in untranslated regions (UTRs). [35] This has allowed for development of models to predict the degree of severity by adjusting the age of patients and analyzing their viral sequences (https://covidoutcome.com/, accessible from 27 December 2021).

It has been proposed that mutations in ORF1a, ORF1b and in genes encoding N protein were related to a high prevalence of asymptomatic scenarios. However, when D614G, Q57H (ORF3a) and S194L (N protein) changes were present, they were associated with mild and severe outcomes. Likewise, a single nucleotide change (nt14408) in RdRp was associated with severe cases of the disease [36].

Regarding prolonged viral RNA shedding, which can be up to 100 days in patients with severe disease, one study reported that viral shedding time decreases when A1,430G or C12,473T mutations are present and increases when G227A is present (*p* < 0.05). Likewise, mutations in G227A, C7,392T, C15,324T, and C25,626T were mostly represented in severe disease cases [47].

The analysis of SARS-CoV-2 variants and their impact on clinical outcomes must be seen from an integral perspective; thus, the different levels of structural organization that make up the variants must be evaluated. In this context, it was determined that three structural changes at the RNA and protein levels, specifically A26194T (T268S) and C25611A (synonymous mutation) in the ORF3a region and C28854T (S194L) in the N protein were associated with an increase in severe cases and fatal outcomes (*p* < 0.05) [22].

Methylation at the m6 A loci of the spike protein has been identified in patients debuting with gastrointestinal symptoms, which could provide underlying mechanisms for its change in virulence and transmission capacity during outbreaks and affect the outcome for serious and severe disease [21].

### 3.3. Rise and Spread of Variants of Concern

As more functional changes in the SARS-CoV-2 sequence were reported, some variants began to be identified by different surveillance systems due to the speed with which their presence was increasing. This has been a cause for concern because it in unknown what effect these changes may have on clinical outcomes, diagnoses and vaccine efficacy [23,29]. The differences of some varieties of the virus from the second half of 2020, their rapid spread, as well as the lack of a clear notation for their classification make it necessary to define the VOCs [13,15,29].

Chronologically, the reported VOCs in the studies analyzed were:

Beta (B.1.351): it was first documented in May 2020, in addition to the D614G substitution, this variant presents other changes such as E484K and N501Y that confer the capacity of immune escape by effect of previous infection or vaccination; the increase in its transmission has been estimated at around 50% compared to the Wuhan variant [30].

Alpha (B.1.1.7): identified in September 2020, presents a 70% increase in transmissibility, consequence of key changes, specifically in the RBM (N501Y) and near the furin cleavage site (P681H), which could increase the affinity for ACE2 and have an impact on infection and transmission, respectively; [30] this could have contributed to the rapid dispersion and dominance of this variant in the world before the arrival of the Delta variant (B.1.617.2). [11,50,51].

Delta: identified in October 2020, it has become the most common variant globally, its main changes are D614G, E484Q and L452R, it has been reported that this variant has biological and clinical implications such as increased risk of hospitalization, longer duration of virus release by infected persons, low Ct values in PCR, greater affinity to the ACE2 receptor, mechanisms of escape to the effect of antibodies and transmissibility increased by 50% [50,51].

Gamma (P.1): first documented in November 2020, highlighting the presence of three changes that confer affinity for the ACE2 receptor, these are K417T, E484K and N501Y which contribute to its increased transmissibility estimated at 40% in relation to the first variants [30].

When independently analyzing the clinical outcomes associated with VOC, it was identified that the Alpha and Delta variants affect individuals with similar demographic and comorbidity characteristics, while patients infected with the Gamma variant are older people, mainly between 45 and 64 years old, with a higher probability of presenting cough and anosmia, compared to the other variants [29,30].

One of the largest studies conducted to date, focused on determining fatal outcomes and admission to intensive care unit (ICU), showed that people infected with the Alpha variant had a higher risk of admission to ICU and 28-day mortality compared to those infected with other unrelated lineages [13]. Likewise, Veneti et al., [49] analyzed 23,169 cases of infected individuals with the Alpha and Beta variants as well as other non-VOCs and determined that these two VOCs were associated with an increased risk of hospitalization and ICU admission.

The analysis by sex has documented significant differences in clinical outcomes associated with variants. In this regard, a study reported that women infected with the B.1.1.7 lineage develop a more severe disease compared to men, as well as women infected with other lineages, these outcomes are associated with admission to ICU, as well as a slight risk of mortality [11].

One study concluded that the Delta variant, after adjusting age and sex, was associated with increased oxygen requirement, admission to ICU, and death when compared with Alpha and Beta. It was also reported that this variant was associated with increased viral loading, as well as prolonged viral shedding [50]. In contrast, another study noted that the presence of this variant in different parts of the United States of America did not result in increased hospitalizations, ICU admission or death in adults. Partly, impact of this variant on transmission rates and fatal outcomes was associated with people who had not yet been vaccinated, including adults younger than 50 years of age [51].

### 3.4. Other Variants Related with Clinical Outcomes

The dynamics of the SARS-CoV-2 variants analyzed throughout the pandemic has been complex. In France, after the first outbreak there were new variants that had an epidemiological impact; in the comparative study by Fournier et al. [28] it was determined that the Marseille-4 variant had 13 changes, one of which (S477N) was associated with hypoxemia (*p* < 0.05). This variant could be associated with changes in the affinity for ACE2 and decrease the sensitivity of the virus to neutralizing antibodies. In this same context, a cohort study conducted in France determined that lineages B.1.177 and B.1.160, Marseille-2 and Marseille-4, respectively, during the second phase of the pandemic, were associated with more severe clinical outcomes and consequently higher mortality and hospitalization rates [29], however in this study the association between variants and disease severity was not clear. 

Conversely, the B.1.243 lineage was found to be significantly associated with a high degree of disease severity and fatal outcomes. This lineage shows several substitutions in NSP12:P323L, N:S194L as well as D614G and P681H changes in the spike protein [30].

The B.1.616 lineage whose differences from the original SARS-CoV-2 are centered on nine changes and one deletion in the spike protein (H66D, G142V, Y144del, D215G, V483A, D614G, H655Y, G669S, Q949R, N1187D), as well as changes in other regions, was associated with a high 28-day fatality rate when compared to VOC and other unknown lineages (*p* < 0.05) [32].

Conversely, when analyzing the degree of disease severity with SARS-CoV-2 variants, Al Khatib et al. [27] identified changes in specific regions of the B.1 and B.2 lineages associated with severe symptoms; patients who developed worse clinical scenarios had greater variability in the SARS-CoV-2 analyzed sequences (*p* value 0.001).

When different clades were analyzed with respect to their clinical outcomes, it was determined that the L/V clades (variant of the ORF3a coding protein NS3-G251) were associated with more severe outcomes as they had more pronounced systemic inflammation with higher concentrations of proinflammatory cytokines, chemokines and growth factors compared to the G, S and O clades [46]. Conversely, when outcomes were analyzed with respect to infection by the G and S/L clades, it was observed that, regardless of clade, the results were similar in terms of rate of hospitalizations and death [39]. One study reported that clade V was statistically related to increased mortality in uni- and multivariate analyses compared to other variants [42].

It has been reported that the M1V variant has lower rates of dyspnea, rhinitis and hospitalizations, which has been related to its infection in younger age groups, while the M4V variant infects mainly older adults and has a higher probability of producing fever, lower frequency of cough, rhinitis and olfactory and gustatory disorders, as well as a higher rate of hospitalization associated with hypoxemia. It has also been noted that the M4V variant confers some immunological escape and has been the responsible for cases of reinfection [28,48].

### 3.5. Critical Appraisal of the Studies

There was a heterogeneous presentation in the articles analyzed in terms of study design, SARS-CoV-2 variants, and specific description of clinical outcomes. The selected studies were appraised using the Newcastle–Ottawa Quality Assessment Scale checklists adapted for cross-sectional and cohort studies [19,20]. Regarding cross-sectional and cohort studies, four (12.12%) were scored as satisfactory, fifteen (45.46%) were scored as good and one (3.03%) was scored as very good. The rest of the studies included ten (30.30%) experimental and one (3.03%) mathematical modeling analysis, and two (6.06%) short communications were restricted to the description of their limitations (Table 3).

## 4. Discussion

Different variants of SARS-CoV-2 have emerged from geographic regions whose epidemiological conditions allowed for the stabilization of certain genetic combinations that had an impact on their fitness. It has been proposed that the origin of SARS-CoV-2 variants are hosts with long periods of infections, as people with cancer or immunocompromised condition, as well as uncontrolled circulation in countries with poor health infrastructure, which when added to selection pressures, has favored the adaptation and dominance of new lineages [7,30,40,43,50,52].

Studies in SARS-CoV-2 have focused on the spike protein (Table 1 and Figure 2), changes in its sequence have been associated with increased affinity for ACE2, immune escape and increased infectivity and transmissibility [7]. In evolutionary terms, this suggests that there are specific regions that are susceptible to accumulate mutations under positive selection, regardless of lineages of origin. However, the sites that may have an impact on severity, as well as on the emergence and evolution of new variants, are not circumscribed to a single protein (Table 1). It has been pointed out that, in parallel to local and global epidemiological contexts, the D614G substitution in the spike protein, as well as the R203K and G204R in the N protein, have been important in increasing fitness for SARS-CoV-2 [53].

SARS-CoV-2 variants are characterized by mutations in their genome, with respect to the original strain, understanding at molecular level the impact of these variants will improve our understanding of their mechanisms of infection [54,55]. In this scenario, D614G substitution prevents the interaction of a hydrogen bond with the T859 residue of an adjacent protomer of the spike protein trimer; this leads to a conformational change in the RBD to an “up” conformation, which promotes greater binding to ACE2; it has been mentioned that this promotes greater infectivity of the virion, which has been verified in experimental studies [56,57,58]. Regarding the P681H substitution, this is part of a proteolytic cleavage site for furin and furin-like proteases; it has been pointed out that, in parallel to the cleavage of arginine-rich multi-basic motifs, there may be a preference for other basic residues such as histidine, which could favor the cleavage of S1/S2 in the spike protein and impact the infectious capacity of SARS-CoV-2. [59]. Conversely, it has been reported in an in silico study that the histidine residue shortens the distance by 2 Å with respect to proline, which could promote the binding of spike protein with Neuropilin 1, this protein is a co-receptor for SARS-CoV-2 in cells of the central nervous system (CNS), [60]. Since the onset of the COVID-19 pandemic, the emergence of new variants has been a global concern. However, association studies of clinical outcomes with SARS-CoV-2 variants have been scarce compared to the magnitude of the pandemic [14,15]. Some of them have reported preliminary results in small populations and there has been a lack of reproducibility in other clinical scenarios. Although statistically significant associations between outcomes and variants have been reported [24,27,32,44], these are inconclusive and in some scenarios such associations have been opposing [50,51]. Likewise, it has been identified that not all variants impact disease development (Table 2).

Different clinical outcomes may be linked to genetic variations in SARS-CoV-2. However, it is necessary to adjust for the presence of individual risk factors in order to reliably establish such association (Table 2). In this context, most severe outcomes are associated with pre-existing diseases. Age and the presence of comorbidities such as hypertension, obesity, cardiovascular disease, immunosuppression, smoking, and diabetes mellitus are more important predictors of severity, hospitalization, and mortality than SARS-CoV-2 variants [6,23,39,40,43,61,62]. In the same way, the interaction of different SARS-CoV-2 variants with hosts is bidirectional. Different human polymorphisms have an impact on clinical outcomes: sequence changes in *ApoE*, *TLR7*, *TMEM189-UBE2V1*, as well as *SLC6A20*, *LZTFL1*, *CCR9*, *FYCO1*, *CXCR6*, *XCR1*, have been associated with severe disease outcomes as well as respiratory failure [12,63].

Notwithstanding the above, there are associated SARS-CoV-2 variant phenotypes that have significantly driven the course of the COVID-19 pandemic. This has been widely reported for COVs, in terms of transmissibility and potential evasion of neutralizing antibodies after vaccination or infections [7]. In this regard, the impact that new variants have on the reinfection of individuals has been documented, and some of them produce more severe disease than a first infection [64,65,66,67,68].

Several studies have been displaced as the pandemic progresses and new variants are reported, we have not yet been able to adapt our studies to such rapid changes according to the dynamics of the pandemic and the information that is generated every day, variants that could be considered of interest or have an impact on clinical outcomes, are quickly replaced by others that presented greater fitness [52]. Faced with such changes and the rapid emergence of variants, compared to our capacity to respond, we must seek approaches focused on anticipating future scenarios and not just reacting to established contexts. The Omicron variant (B.1.1.529), with more than 30 changes in the spike protein, as well as in other parts of its sequence, is an example of this; its accelerated infection rate in the world suggests a high capacity to reinfect people who have recovered from other variants such as Delta or to infect individuals with three-dose vaccinations, showing its capacity to evade immune responses and generate clinical outcomes different from those of other variants [69,70,71]. The course of the disease in patients infected with this variant, relative to previous waves, has been mild, with a significantly lower risk of hospitalization, severe disease, and ICU admission or death. However, it has not been clearly established whether this behavior is due to a lower pathogenicity of this variant or to pre-existing immunity [72,73,74].

As for the incubation period, it has been estimated that it may be shorter, around 2 to 3 days. The usual symptomatology is given by upper respiratory tract affection, which makes it difficult to differentiate from the common cold. [75,76,77]. It should be considered that although this variant does not present severe symptoms in a significant proportion of infected persons, the demand for care is high, such that detection and care capacity may be overwhelmed and primary care contact in health systems may collapse. The emergence of variants is an event that will continue to be repeated as time progresses. It has been proposed that in the face of new phases of the pandemic, coordinated approaches are required where global epidemiological surveillance and phenotypic characterization of new variants are linked [78]. However, this represents a challenge because in poor or emerging economy countries, variant sequencing may not be a priority, which coupled with low vaccination rates and lack of follow-up of sanitary measures, represents a potential risk for the emergence of new VOCs [79,80]. 

As data on new SARS-CoV-2 variants become available, more associations can be established on their clinical outcome. However, these results need to be validated with other studies, in particular, those performed in vitro or in silico, and in observational studies where there was no adequate control of biases, which can lead to over-interpretation of results, affecting the degree of validity, reproducibility and reliability of these [81,82,83].

Regarding the critical appraisal of the analyzed articles, most of them were cross-sectional and cohort studies, the sample size was heterogeneous, with a wide range, from 17 to 202,692 participants; their rating according to the Newcastle–Ottawa Quality Assessment Scale checklists was globally adequate (Table 3). Some studies were centered on small sample sizes, the lack of predictive models for disease progression, the use of a database to collect information without sampling specifications on factors of interest such as age, gender, ethnicity or population group, and without complete information on the clinical course or outcome of the disease, which in turn conditioned the presence of some selection and information biases, mainly in the sampling or in the available data to establish associations; in some studies, a low representation of SARS-CoV-2 variants was identified because the population was restricted to captive groups, as in the case of hospitalized patients.

### Limitations

It has been pointed out that different clinical outcomes can be associated with the same variant and therefore, this places into context, the plasticity of virus–host interactions; thus, it is difficult to establish a univocal and generalized association between SARS-CoV-2 variant and clinical outcomes [33,44,47]. Several models focused on measuring the association between disease severity and variant type have shown that once individual variables such as age, sex, ethnicity and comorbidity are neutralized, there is no significant difference in disease severity between variants. It has even been shown that they are not associated with increased hospital admissions, the latter being mostly associated with a higher viral load than with the infection variant itself [6,43,62]. Of the 15 articles that found statistically significant associations between SARS-CoV-2 variants and clinical outcomes, adjustments between various confounding variables were reported in only 8 of them (Table 2). For this reason, more studies are required to understand as a whole the influence of the different variables that impact on clinical outcomes [13,14].

The dynamics and fixation of new SARS-CoV-2 variants around the world has been rapid; several recently published studies focus on variants that have been displaced by other new ones, but that at the time were relevant for clinical outcomes [11], which shows that static scenarios for SARS-CoV-2 do not exist. Some of the information presented in this systematic review could become outdated in a short time; this has occurred in other diagnostic and therapeutic contexts due to the advances of the pandemic around the world and the accumulation of new knowledge related with COVID-19. [78,84,85,86].

This review highlights the impact of SARS-CoV-2 variants and clinical outcomes. Cross-sectional and cohort studies have undergone critical appraisal using an adapted appraisal tool; however, the rest of the studies were highlighted to their limitations, which should be viewed with caution. The analyzed articles were heterogeneous methodologically; some failed to mention potential confounding factors and to describe methods to control them (Table 2 and Table 3). In this context, the analyzed studies make a quantitative analysis or meta-analysis unfeasible. 

Some of the clinical outcomes presented in the reviewed articles did not conform to common outcome measures for the clinical follow-up of the disease [33,87]. It is important to adhere to these measures to identify clinical scenarios of relevance and to propose systematized responses to a pandemic that is far from over. It is recommended that authors who wish to establish associations between clinical outcomes and new variants be more exhaustive reporting these outcomes to cover various aspects associated with infections caused by SARS-CoV-2.

The limitations of this study were centered during the search period; the Omicron variant was announced by the WHO one day after the information was collected for this article; thus, its inclusion was not contemplated in this work. The search algorithm may have been biased in terms of specific searches for information related to clinical outcomes, since there may have been important outcomes that did not fit the algorithm and therefore were not included. Likewise, consulting two databases could have influenced the inclusion of new reports that could potentially increase our knowledge on the topic addressed in this systematic review.

## 5. Conclusions

The most identified SARS-CoV-2 variants in this study presented changes in the spike protein, N protein, RdRp, NSP3, as well as in different ORFs sequences. In most of the analyzed articles, possible associations between SARS-CoV-2 variants and clinical outcomes were found. However, only eight articles reported significant associations adjusting for age, sex, comorbidities, and other variables. There are multiple factors, such as age and pre-existing diseases, involved in the course of COVID-19 disease, that have been determinant in the degree of severity. Nevertheless, the association between variants and clinical outcomes has not been fully explored at present; more research is required to establish possible associations between SARS-CoV-2 variants and illness behavior.

## Figures and Tables

**Table 1:** Changes in SARS-CoV-2 sequences reported in the studies. **^¤^** Changes in nucleotide sequences.

Changes	Location	Sources
▪ D614G, V16F, V367L, K558N, Q675H, A879V, L452R, S939F, V1176F, K1191N, G1219V, S399P, L54F, N501Y, E484K, S477N, L5F, V213A, S689R, A570D, T716I, S982A, D1118H, P681H, N439K, V83L, W258R, Q677H, N811I, S640A, V6FS, H66D, D215G, V483A, H655Y, G669S, Q949R, and N1187D. ▪ m6 A methylation. ▪ Non-synonymous 21,575; 25,106; 23,403; 24,099, and 24,453. ^¤^ ▪ Deletion 21,603–21,614. ^¤^	Spike protein (S)	[6,21,23,24,25,26,27,28,30,31,32,33,34,35,36,39,40,41,42,43,44]
▪ R203K, I292T, G204R, S202N, M234I, A376T, S194L, P13L, A119S, Q160R, S193I, R195S, P199S, V30L, G212V, and S197L.	Nucleocapsid phosphoprotein (N protein)	[22,24,28,30,33,35,36,43,44]
▪ L3606F, and C370R. ▪ Synonymous 19,944, and 20,764. ^¤^ ▪ Insertion 11,074. ^¤^	ORF1a	[22,27,32]
▪ A138T.	NSP1	[44]
▪ T85I, A205V, V247A, T256I, Q321K and T814I.	NSP2	[30,33,39,44]
▪ F106F, P822L, P679S, T1022I, A1179V, T1198K, F1354C, P1665L, L916, F924, D1585, N1673, and 8782C.	NSP3	[22,24,33,39,41,42,44]
▪ F308Y, S76S, A231V, E3073A, and A323S.	NSP4	[24,33,35,39,41,44]
▪ E3909G.	NSP7	[44]
▪ A21T and T4040I.	NSP8	[33,44]
▪ L42F.	NSP9	[33]
▪ A176S, P314L and V767L.	NSP12	[22,28,41]
▪ P504L, Y541C, T127I, T153I, V169F, M576I, S5398L, and P203L.	NSP13	[33,39,41,44]
▪ L7L.	NSP14	[39,41]
▪ H337Y.	NSP15	[33]
▪ Y222C.	NSP16	[33]
▪ G251V, G196V, S253P, Q57H, A54V, A99S, T151I, and D222Y. ▪ Deletion 25,710–25,715. ^¤^	ORF3a	[22,24,28,30,33,35,36,39,42,43,44,45]
▪ I33T.	ORF6	[24,43]
▪ Deletion 27,508–27,751. ^¤^	ORF7b	[43]
▪ L84S.	ORF8	[23,24,35,36,39,41,45,46]
▪ A97V, P323L, P232L, P227L, T248I, A656S, H892Y, M906V; G227A; C865T; Y4424; P4715L, 14408C, and C14408T. ▪ Nucleotic substitution nt14408	RdRp	[22,23,24,25,27,30,33,35,36,39,42,43,44,47]
▪ G3728S.	3C-like protease	[44]

**Table 2:** SARS-CoV-2 variants and clinical outcomes. ^‡^ statistically significant; ^§^ non-statistically significant; ^¥^ possible associations; ND, not determined.

SARS-CoV-2 Variants Analyzed	Clinical Outcomes (Main Findings)	Adjustment	Source
▪ Clades S and G (GH and GR) vs. Wuhan-Hu-1	D614G and clade G are not associated with any clinical parameter, severity or lethality. ^¥^	Age, sex and comorbidities	[23]
▪ Clade G: D614G (spike protein).	No association between D614G and crude case fatality rate. ^‡^	ND	[25]
▪ 614G vs. 614D (spike protein) variants.	No evidence of association between the 614G and 614D variants and clinical outcomes. ^‡^	Age, sex, comorbidities	[26]
▪ N439K (spike protein) variant.	No changes in clinical outcomes. ^‡^	ND	[31]
▪ N501Y (spike protein) variant.	No increase in fatality risk. ^‡^	ND	[34]
▪ Variant with changes in ORF1a, ORF1b, ORF10, spike protein, ORF3a, ORF8, N protein and ORF10.	Asymptomatic disease. ^‡^	Age and sex	[36]
▪ Variant with changes in spike protein, ORF1a, ORF1b, ORF8 and N protein	Mild/Moderate disease. ^‡^		
▪ Variant with changes in spike protein, ORF1a, ORF3a, N protein and RdRp.	Severe disease. ^‡^		
▪ Clade L, GH, GR and O.	No significant association in clades with changes in S protein with hospitalization or mortality. ^§^	Age and comorbidities	[40]
▪ Clade 1 (V, S, L and Wuhan) and Clade 2 (G, GR, and GH)	Hospitalization and ICU admission was similar between clades. Variants containing D614G change were associated with increased survival in hospitalized patients. Clade V was statistically related to increased mortality. ^‡^	Age, sex and comorbidities	[42]
▪ B.1 and B.1.1 lineages, changes in 5′UTR region, ORF1a, ORF1b, N protein, ORF6 and spike protein.	No changes in clinical outcomes. ^‡^	ND	[43]
▪ A1,430G o C12,473T and G227A variants.	G227A increase prolonged viral RNA shedding. ^‡^ G227A, C7,392T, C15,324T, and C25,626T related with severe outcomes. ^‡^	ND	[47]
▪ Clade G: D614G (spike protein).	Potential increase in severity in infected individuals. ^¥^	ND	[6]
▪ B.1.1.7 lineage.	Increased clinical severity is not concluded. ^‡^	ND	[15]
▪ ZJ01variant (spike protein).	m6 A methylation possibly related to gastrointestinal symptoms. ^¥^	ND	[21]
▪ Clade GH, GR O and L.	In clade GH V213A in spike protein was possibly associated with fatal outcome. ^§^	ND	[33]
▪ Variants with changes in D614G (spike protein), F106F (NSP3), P314L (NSP12b), 5′UTR, S76S (NSP4), L84S (ORF8), L7L (NSP14), Y541C and P504L (NSP13).	Y541C and P504L variants associated with possible infection and mortality rates. ^‡^	ND	[41]
▪ Variants with multiple changes in NSP4, NSP6, NSP7, NSP8, NSP13, spike protein, RdpRp, N protein and 3C-like protease.	L3606-Nsp6 deletion, S5398L and E3909G-Nsp7 were linked to a higher prevalence of cough and conjunctival congestion, increased risk of fever duration and progression and shorter duration of symptoms, respectively. ^‡^	Age, sex and comorbidities	[44]
▪ Variant with changes in L54F, D614G and V1176F (spike protein), A97V and P323L (RdRp), Q57H and G251V (ORF3a), P13L, S194L, R203K, G204R and I292T (N), I33T (ORF6), S1197R and T1198K (NSP3).	Hospitalization and severe disease. ^‡^	ND	[24]
▪ Variant with changes in L84S (ORF8), G196V (ORF3a), L37F (NSP6), F308Y (NSP4) and S197L (N protein)	Mild disease. ^‡^		
▪ Variant with changes in L71F (NSP7) and S253P (ORF3A)	Fatal outcomes. ^‡^		
▪ Clade 1 (GH/20C, G/20A and G/20B) vs. Clade 2 (S/19B and L/19A).	Trend toward higher rates of hospitalization in clade 2. ^§^ Viral clade contributes minimally to clinical outcome. ^¥^	Age and comorbidities	[39]
▪ Variant with changes in L84S (ORF8) and G251V (ORF3a).	Mild disease. ^§^	ND	[45]
▪ Clade S, Clade O (B.6) vs. Clade L/V	Clades S and O were associated with mild and attenuated disease, respectively. ^‡^ Clades L/V were associated with higher concentrations of cytokines, chemokines and growth factors related to lung injury and regeneration, severe outcome. ^‡^	Age and sex	[46]
▪ Clade G (GH and GR)	Associated with lower risk of severe disease and transmissibility. ^‡^		
▪ M1 variant vs. Clade 20AS	Lower rates of severe disease and hospitalization in clade 20AS. ^‡^	Age, sex and comorbidities	[48]
▪ B.1.160 lineage vs. M1 variant and Clade 20AS	Tendency to mild disease in MV4. ^‡^		
▪ B.1.160 lineage vs. Clade 20AS.	Lower rates of mild disease in MV4. ^‡^		
▪ B.1.160 lineage vs. M1 variant.	Higher rates of hospitalization in MV4. ^‡^		
▪ N501Y variant vs. Clade 20AS and B.1.160 lineage.	Lower rates of hospitalization in N501Y. ^‡^		
▪ B.1.1.7 lineage vs. non-B.1.1.7.	Severe disease in women associated with B.1.1.7. ^‡^	Age, sex, ethnicity and number of comorbidities	[11]
▪ B.1 and B.2, lineages. Multiple changes in ORF1a, spike protein, ORF3a, ORF1b, N protein, ORF14, OFR8 and presence of indels.	Higher number of changes in RNA and protein sequences in patients were associated with severe symptoms compared to mild, especially in elderly. ^‡^	ND	[27]
▪ B.1.160 lineage vs. Clade 20A.	Trend toward hypoxemia in MV4 due to short protective immunity or a lack of cross-immunity. ^‡^	ND	[28]
▪ Clade 20A, Clade 20B, Clade 20C, B.1.177, B.1.160, B.1.1.7, B.1.351, P.1 and A.27 lineages.	B.1.177 and B.1.160 variants were associated with more severe outcomes, including mortality compared with the other variants and clades. ^¥^	ND	[29]
▪ V1176F, S477N and DG14G (spike protein) and S194L (N protein) variants vs. Wuhan strain	Severe disease was statistically associated with changes in spike protein. ^‡^	ND	[35]
▪ B.1.1.7, B.1.351 lineages and non-VOC lineages.	B.1.1.7 and B.1.351 were associatedwith increased risk of hospitalization and intensive care unit (ICU) admission. ^‡^	Age, sex and number of comorbidities	[49]
▪ B.1.1.7, B.1.351, B.1.617.2 lineages and non -VOC lineages.	B.1.617.2 was associated with higher odds or oxygen requirement, ICU admission or death. ^‡^	Age, sex, comorbidities and vaccination.	[50]
▪ B.1.617.2 lineage.	No differences in hospitalization rates, ICU admission and mortality. ^‡^	ND	[51]
▪ B.1.1.7 lineage vs. non-B.1.1.7.	Higher risk of ICU admission and mortality were associated with B.1.1.7. ^‡^	Age, sex, ethnicity, BMI and severe comorbidities	[13]
▪ Clades 19A, 20A and 20B: multiple changes located in ORF1b, spike protein, ORF3a, ORF1a, N protein and NSP3.	Changes in A26194T, C25611A, C28854T and TG11082T were associated with higher rates of severe disease and mortality. ^‡^	ND	[22]
▪ B.1.2, B.1.1.7, B.1.243, B.1.596 and B.1.526.1 lineages.	B.1.243 was associated with severe or fatal outcome. ^‡^	ND	[30]
▪ B.1.616 vs. B.1.1.7, B.1.351 and P.1 lineages.	Severe disease and lethality were associated with B.1.616. ^‡^	Age, comorbidities and healthcare-related COVID-19	[32]

**Table 3:** Critical appraisal of selected studies. ^§^ The Newcastle–Ottawa Quality Assessment Scale checklists adapted for cross-sectional and cohort studies. ND, not determined. * Asterisks correspond to ratings assigned for each item according to The Newcastle–Ottawa Quality Assessment Scale.

Critical Appraisal of Selected Studies							
Source	Design Study	Sample Size	Limitations	Appraisal ^§^			
				Selection	Comparability	Outcome	Score
[21]	Cross-sectional	651 patients	Lack of predictive model for disease progression	****	*	**	7
[24]	Cross-sectional	4556 patients	Sampling bias, database with general information and cofounding factors	***	**	**	7
[39]	Cross-sectional	190 patients	Cofounders and data limited to hospitalization and death and not generalizable to others geographical regions or health systems	****	**	**	8
[40]	Cross-sectional	51 patients	Small sample size and analysis limited to some mutations and prognostic factors	***	**	***	8
[42]	Cross-sectional	302 patients	Underrepresented clades	****	**	***	9
[33]	Cross-sectional	17 patients	Small sample size and underrepresented clusters	***	*	**	6
[15]	Cross-sectional	1479 patients	Retrospective incomplete data and small sample size	***	*	**	6
[51]	Cross-sectional	7615 patients	Undercounted hospitalizations and population groups and small sample size	***	**	**	7
[27]	Cross-sectional	46 patients	Small sample size restricted to some age groups and levels of disease severity	***	*	**	6
[43]	Cross-sectional	71 participants	Heterogeneity in age and disease severity	***	**	**	7
[44]	Cross-sectional	50 patients	Underestimated geographical and density viral distribution	***	**	**	7
[46]	Cohort	319 patients	Selection and information bias	***	**	***	8
[48]	Cohort	740 patients	Missing data and convenience sampling	***	**	***	8
[29]	Cohort	1760 patients	Selection bias	***	**	***	8
[49]	Cohort	28,301 patients	Selection and information bias	***	**	**	7
[50]	Cohort	1675 patients	Small sample size and selection bias	***	**	**	7
[47]	Cohort	43 patients	Small sample size, lack of paraclinical information in severe/critical cases and measurement bias	****		**	6
[13]	Cohort	202,692 patients	Selection bias, cofounders and restricted analysis	****	**	**	8
[11]	Cohort	2341 patients	Sample restricted to hospitalized patients and not generalizable to the population and lack of information about patients’ vaccination status	****	**	**	8
[32]	Cohort	114 patients	Small sample size, retrospective design and selection of controls	****	**	*	7
[22]	Experimental	196 participants	Limited data due to the need of an integrative analysis in which clinical and genetic components of the disease are co-analyzed	ND			
[36]	Experimental	1329 SARS-CoV-2 genome sequences	Lack of correlation in broader clinical scenarios	ND			
[25]	Experimental	2803 SARS-CoV-2 genome sequences	Lack of clinical metadata, sampling bias and heterogeneity in population demographics, testing, definitions and measurements	ND			
[35]	Experimental	9781 SARS-CoV-2 genomes sequences	Limited data due to statistical associations that should be confirmed by other studies	ND			
[31]	Experimental	442 samples	Limitation in data collection	ND			
[23]	Experimental	44 SARS-CoV-2 genome sequences	Limited phylogenetic, phylogeographic and clinical correlation analyses	ND			
[28]	Experimental	1038 SARS-CoV-2 genome sequences	Limited to a single geographic area	ND			
[30]	Experimental	1600 SARS-CoV-2 complete or near-complete genomes	Small sample of severe cases restricting adjustment and analysis of good quality genomes	ND			
[26]	Experimental	3940 genomes sequences	Sampling bias	ND			
[41]	Experimental	1962 genome sequences	Lack of analysis of covariates	ND			
[34]	Experimental	149,789 SARS-CoV-2 genomes sequences	Intrinsic biological mechanisms, co-mutations and lack of individual patients´ information	ND			
[6]	Short communication	4246SARS-CoV-2genome sequences	Limited data due to absence of experimental studies	ND			
[45]	Short communication	11970 SARS-CoV-2 genome sequences	Limited data to establish associations	ND			
